# Effects of shared decision-making on the prognosis of peritoneal dialysis patients

**DOI:** 10.1097/MD.0000000000040659

**Published:** 2024-11-22

**Authors:** Byung Hwa Park, Ho Sik Shin, Jinseog Kim, Jeonghwan Lee, Ji Hyeon Park, Gang Jee Ko, Won Min Hwang, Do Hyoung Kim, Young Ki Lee

**Affiliations:** a Department of Internal Medicine, Renal Division, Gospel Hospital, Kosin University College of Medicine, Busan, South Korea; b Transplantation Research Institute, Kosin University College of Medicine, Busan, South Korea; c Department of Bigdata and Applied Statistics, College of Science and Technology, Dongguk University, Gyeongju, Republic of Korea; d Department of Internal Medicine, Seoul National University College of Medicine, Seoul, Republic of Korea; e Department of Internal Medicine, National Police Hospital, Seoul, Republic of Korea; f Department of Internal Medicine, Korea University College of Medicine, Seoul, Korea; g Department of Internal Medicine, Konyang University Hospital, Daejeon, Republic of Korea; h Department of Internal Medicine, Kangnam Sacred Heart Hospital, Hallym University College of Medicine, Seoul, Republic of Korea.

**Keywords:** chronic kidney disease, dialysis, prognosis, shared decision-making

## Abstract

**Background:**

Chronic kidney disease (CKD) patients face critical decisions in choosing kidney replacement therapy such as hemodialysis (HD) or peritoneal dialysis (PD), which significantly affect their quality of life and health outcomes. Recent studies highlight the importance of shared decision-making (SDM) in helping patients understand their treatment options and make informed choices. SDM not only improves patient satisfaction and autonomy but also emphasizes the need for comprehensive pre-dialysis education to support optimal treatment selection.

**Methods:**

Among patients with chronic kidney failure from 8 hospitals in Korea who started dialysis, 256 who participated in a pilot project for home management of PD were included in the present study. A mixed-methods study was conducted using questionnaires and semi-structured interviews. Our study focused on the effects of SDM on patient death, survival rate, HD conversion, emergency room visits, hospitalization days, and outpatient visits.

**Results:**

A significant difference was observed in hospitalization days (*P* = .0044) between the SDM and non-SDM groups. However, no significant differences were observed in survival rate, rate of conversion to HD, survival rate after conversion to HD, emergency room visit rate, number of hospitalizations per patient, outpatient visit rate, medical cost, hospitalization cost, outpatient cost, and phosphate-binding agent prescription rate.

**Conclusions:**

This study emphasizes the benefits of SDM in reducing hospitalization days for PD patients, suggesting its potential role as a guide in future decisions regarding PD. PD provides a particularly beneficial home-based treatment alternative for patients facing challenges with hospital visits, supported by advanced technologies. Overseas, various countries are implementing policies and incentives to promote home dialysis, demonstrating the potential for SDM to enhance patient satisfaction and outcomes in dialysis care globally.

**Key points**

- Patients with chronic kidney disease (CKD) must make crucial decisions regarding the choice of kidney replacement therapy (RRT).
- Recent studies have emphasized the importance of shared decision-making (SDM) in helping patients understand their treatment options and make informed choices.
- A study involving 256 patients participating in a pilot project for home management of peritoneal dialysis (PD) in Korea found that SDM significantly reduced hospitalization days (*P* = .0044).
- However, no significant differences were observed in survival rates, conversion rates to hemodialysis (HD), emergency room visit rates, and other indicators.
- This study suggests that SDM is beneficial in reducing hospitalization days for PD patients and may play a crucial role in future PD-related decisions.
- Additionally, policies and incentives promoting home dialysis are being implemented worldwide, indicating that SDM has the potential to improve patient satisfaction and outcomes in dialysis treatment globally.

## 1. Introduction

Chronic kidney disease (CKD) is a condition in which the kidneys gradually lose function, potentially leading to kidney failure if left untreated.^[1,2]^ CKD patients must choose a treatment method to replace their kidney function, which significantly impacts their quality of life and health.^[1,2]^

One of the critical factors, CKD patients must consider when selecting a kidney replacement therapy (RRT) is the type of dialysis. Patients can choose between hemodialysis (HD) and PD.^[1]^ PD is a treatment method that uses the patient’s peritoneum to perform cleaning and ultrafiltration, allowing the patient to perform dialysis at home and ensuring their autonomy.^[3,4]^ Patients undergoing PD need to visit the dialysis clinic only twice a month, although the frequency may vary by hospital.^[5]^

According to the 2021 Korean Society of Nephrology’s report on the current status of RRT in Korea, the proportion of patients undergoing PD has been gradually decreasing compared to HD or kidney transplantation.^[2]^ In 2006, the proportion of PD among all RRT patients was 28%, but it decreased to 4% by 2019. The reasons for this decline include: ① relatively lower insurance coverage compared to HD, ② a shortage of dedicated PD personnel (especially nurses), ③ an increase in the establishment of HD units, and ④ a lack of shared decision-making (SDM) education for choosing the type of dialysis.^[2]^

Recent studies emphasize the importance of providing information during the patient’s treatment selection process.^[6]^ Education and counseling help patients understand their health status and treatment options, leading to more satisfactory decisions.^[6]^ Additionally, it has been reported that providing education before starting dialysis increases the selection of PD.^[7]^

In the process of choosing a treatment method, it is crucial to provide patients with sufficient medical information and counseling.^[8,9]^ The recently highlighted SDM approach enhances such communication and actively involves the patient in their treatment.^[8,9]^

SDM involves healthcare providers and patients sharing decisions about the best dialysis method that aligns with the patient’s values.^[10–12]^ During this process, patients understand the treatment goals and the pros and cons of each option.^[10–12]^ SDM also improves patients’ quality of life and maintains their autonomy.^[13,14]^ International guidelines recommend that all CKD patients receive pre-dialysis education to help them choose an RRT method.^[11,15–17]^ Despite these recommendations, many patients feel unprepared for starting dialysis and uninformed about the available treatment options.^[6,18–20]^ This situation can lead to emergency dialysis or the selection of inappropriate dialysis methods, causing patients to lose the opportunity to make their own choices.^[21,22]^

According to studies conducted worldwide, comparing COVID-19 incidence and mortality rates between HD patients and home dialysis PD patients, home-dialysis PD patients had lower rates of COVID-19 incidence and mortality compared to HD patients.^[23,24]^ Therefore, interest in PD as a safe dialysis method has increased during the COVID-19 pandemic, and there is a need to establish a systematic management system for PD patients’ self-care.^[23,24]^

This study was conducted as part of the PD home management pilot project initiated in 2019, focusing on SDM to determine the type of dialysis. A survey was conducted on patients who received SDM to determine the current status and satisfaction with the SDM process. The study compared emergency room visits, hospitalization rates, hospitalization periods, conversion to HD, mortality data, direct medical costs (total claimable health insurance costs), inpatient and outpatient medical expenses, and the use of phosphate binders and erythropoiesis-stimulating agents between patients who underwent SDM and those who did not. The educational counseling fee I (This applies when doctors explain medical conditions and treatment plans to patients who come to the clinic. The counseling is more detailed and professional, helping patients understand their health better and manage it safely on their own.) and educational counseling fee II (This applies when either a doctor or a nurse provides counseling specifically related to managing PD. It includes teaching both outpatient and hospitalized patients how to care for themselves, prevent complications, and manage their dialysis safely.) checklists used in the study were retrospectively collected from data submitted by institutions registered in the pilot project to the Health Insurance Review and Assessment Service, and statistically analyzed.

This study is the first report on the selection of dialysis methods through the SDM process in Korea. It is expected to contribute to providing a foundation for better treatment decisions for both healthcare providers and patients.

## 2. Method

The purpose of this study is to provide appropriate home medical services to PD patients. It analyzed the effects of the clinical trial pilot project for home management of PD patients since December 16, 2019 to December 31, 2021. In this study, we retrospectively collected and analyzed clinical data and claims data submitted to the Health Insurance Review and Assessment Service pilot project data submission system. Using the results, we prospectively studied to determine the status of the pilot project for home management of PD patients and evaluate its clinical effectiveness.

### 2.1. Study population

It was conducted on 10 hospitals that participated in the pilot project. Among patients receiving PD, 6149 patients were retrospectively examined, excluding those who met the exclusion criteria. Exclusion criteria were as follows:

(1) conditions requiring emergent HD, including uncontrolled severe acute heart failure; New York Heart Association, Grade IV, uremic encephalopathy, severe electrolyte disorders (serum potassium [s − K+] > 6.5 mmol/L), and unstable vital signs.
(2) complications associated with other serious diseases, such as severe systemic infections or sepsis.
(3) refusal to participate in the study
(4) participation in other clinical studies.

2989 patients were unregistered patients and 2429 patients were already receiving PD before the study in 83 hospitals. The lists of hospitals are as below.

Here are the translations of the hospital names (see Supplementary 1, Supplemental Digital Content, http://links.lww.com/MD/O23).

**Figure 1.**
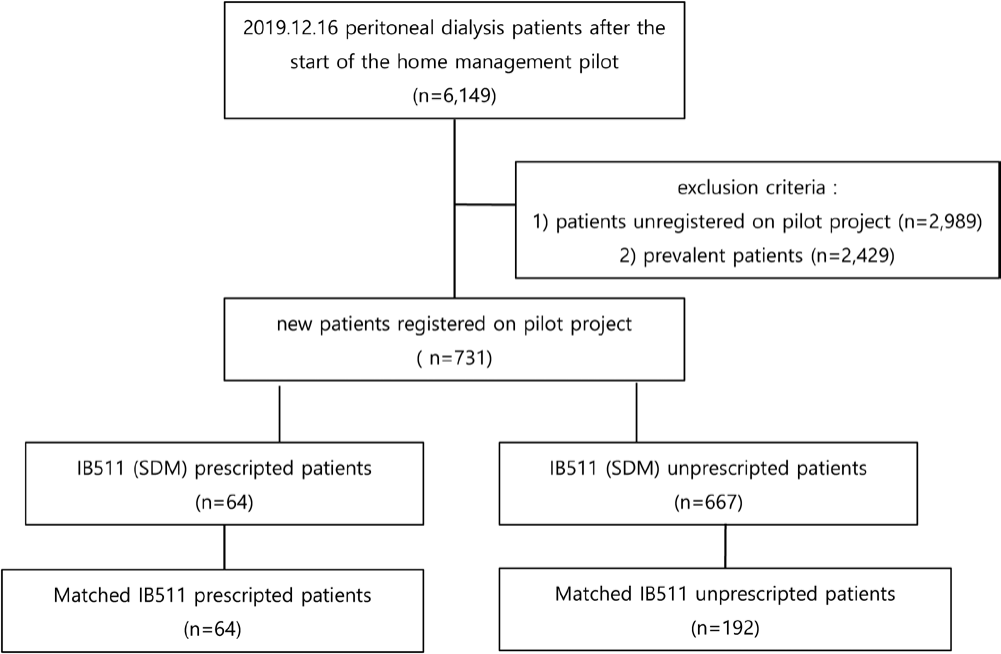
Effect of SDM on patient’s prognosis. SDM = shared decision-making.

Total 731 patients were studied retrospectively (Fig. 1). Even if there was a difference in underlying disease between the patient groups, there was no difference in the level of consciousness to determine the SDM process.

The target patients were divided into the SDM group (patients with educational counseling fee I (IB511) prescription) and the control group (patients without educational consultation fee I (IB511) prescription), and propensity score matching (age, sex, diabetes status, and type of insurance) was utilized to determine the patient group. Among 731 patients, 64 patients had SDM prescription and 667 patients had not SDM prescription as a control group. After that, 475 patients out of the 667 patients control group who met the exclusion criteria were excluded. Finally, 64 patients had SDM prescription and 192 patients had not SDM prescription. Patient group and control group matched at a ratio of 1:3. Patient mortality, survival rate, HD conversion, emergency room visits, hospitalization days, and outpatient visits were compared and analyzed in both groups (Fig. 1).

### 2.2. SDM study setting

Patient education program proceed to depending on the stage of CKD. In particular, when CKD progressed to stage 5, the SDM process proceeded as follows; First, the study provides prior education once to patients who need SDM by providing them with educational materials (educational booklets comparing various dialysis methods and video materials introducing SDM, HD, and PD) to help them make dialysis decisions. Second, medical staff (doctor and nurse) and patients share opinions on types of dialysis based on prior training. Third, medical staff and patients share opinions about options for dialysis methods considering situation (residential area, job, and lifestyle) of patients. Fourth, medical staff and patients share opinions to decide on the type of dialysis. If the decision is difficult, it will be decided at the next outpatients visit.

A survey was conducted during this SDM process to investigate SDM measurement scale, patient satisfaction scale, and disease perception scale (see Supplementary 2_Survey, Supplemental Digital Content, http://links.lww.com/MD/O24).

### 2.3. Statistical analysis

All data were analyzed using SPSS. Results are expressed as the mean ± standard error of the mean. Statistical differences among the groups were assessed using the paired *t* test for comparison between 2 groups. Differences were considered statistically significant at *P* < .05. The study focused on the results of clinical indicators; effects of SDM on patient mortality, survival rate, HD conversion, emergency room visits, hospitalization days, and outpatient visits were retrospectively investigated and statistical analysis was performed.

This study was conducted in accordance with the principles of the Declaration of Helsinki. Since the survey was conducted anonymously, approval from the ethics committee is not required.

## 3. Results

In this study, 256 patients were investigated; 64 patients had SDM prescription (SDM group) and 192 had no SDM prescription (non-SDM group). The observation period was longer in the non-SDM group than in the SDM group, and the prescription rate for educational counseling 1 fees was higher (Table 1).

**Table 1:** Characteristics and death, number of HD conversion of patients and according to implementation of SDM.

		Total (256)	Patient group (64)	Control group (192)	*P* value
Observation period (month)		9.5 ± 5.8	7.3 ± 5.1	10.3 ± 5.9	<.0001
Age		52.03 ± 14.59	52.69 ± 13.78	51.81 ± 14.88	.6652
Sex	Female	81 (31.64)	20 (31.25)	61 (31.77)	>.99
	Male	175 (68.36)	44 (68.75)	131 (68.23)	
Insurance	Health insurance	237 (92.58)	58 (90.62)	179 (93.23)	.6796
	Medical benefit	19 (7.42)	6 (9.38)	13 (6.77)	
Underlying disease	Hypertension	251 (98.05)	64 (100.00)	187 (97.40)	.4341
	Diabetes mellitus	155 (60.55)	39 (60.94)	116 (60.42)	>.99
	Ischemic heart disease	77 (30.08)	15 (23.44)	62 (32.29)	.2379
	Stroke	21 (8.20)	1 (1.56)	20 (10.42)	.0486
	Atrial fibrillation	12 (4.69)	3 (4.69)	9 (4.69)	1.0000
Education consulting fee 1 (IB510)		135 (52.73)	23 (35.94)	112 (58.33)	.0030
Education consulting fee 2 (IB520)		214 (83.59)	52 (81.25)	162 (84.38)	.6967
Patient care fee (IB530)		248 (96.88)	62 (96.88)	186 (96.88)	>.99
Death		4 (1.56%)	0 (0%)	4 (2.08%)	.5606
Total observational period (human year)		203.7	38.8	164.9	
Mortality rate (/1000 human year)		19.6	0.0	24.3	
Converting to hemodialysis		36 (14.1)	6 (9.4)	30 (15.6)	.3026

HD = hemodialysis, SDM = shared decision-making.

**Figure 2.**
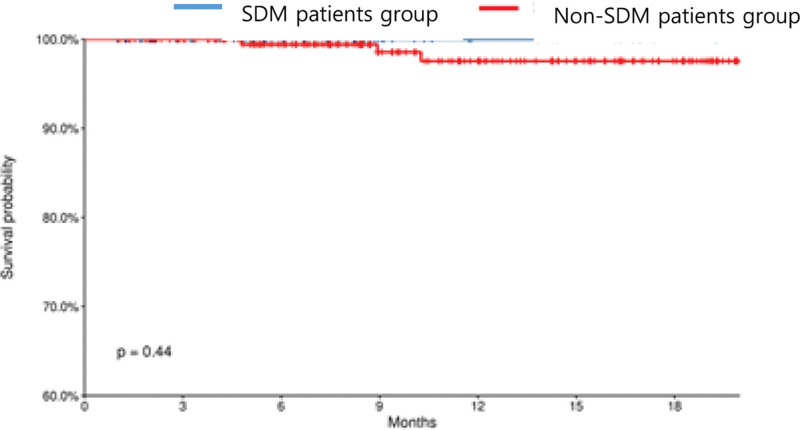
Survival analysis according to patient death according to implementation of SDM. SDM = shared decision making.

Among the patients, 4 deaths occurred in the non-SDM group and none died in the SDM group. The survival rate analysis revealed no significant differences between the 2 groups (*P* = .5606) (Table 1, Fig. 2).

**Figure 3.**
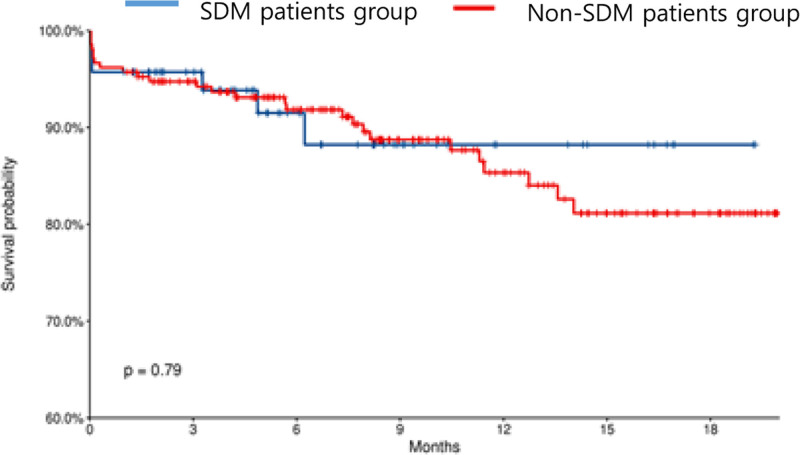
Survival analysis for HD conversion according to implementation of SDM. HD = hemodialysis; SDM = shared decision-making.

Among the analyzed patients, 36 converted to HD (6 in the SDM group and 30 in the non-SDM group), and no significant difference was observed between the 2 groups (*P* = .3026). In addition, no significant difference was found in the survival rate between the 2 groups in terms of changing to HD (Table 1, Fig. 3).

The number of annual emergency room visits per patient was 0.63 in the SDM group and 0.89 in the non-SDM group, and did not significantly differ (*P* = .3574). There was no significant difference was observed in sex, age, and type of hospital between the 2 groups (*P* = .8658, *P* = .3135, *P* = .6790, *P* = .6593, *P* = .2345, *P* = .2726, *P* = .0186, *P* = .6057, *P* = .4208, *P* = .9311, respectively) (Table 2).

**Table 2:** Number of emergency room visits per year, annual number of outpatient visits per patient according to implementation of SDM.

		Emergency room visits per year				Annual number of outpatient visits			
		Total (n = 256)	Patient group (n = 64)	Control group (n = 192)	*P* value	Total (n = 256)	Patient group (n = 64)	Control group (n = 192)	*P* value
Total		0.83 (2.50)	0.63 (1.65)	0.89 (2.73)	.3574*	51.51 (23.06)	51.16 (22.01)	51.63 (23.46)	.8862*
Sex	Female	0.65 (2.70)	0.58 (1.64)	0.67 (2.98)	.8658*	57.54 (27.02)	56.66 (24.85)	57.83 (27.89)	.8610*
	Male	0.91 (2.41)	0.65 (1.68)	1.00 (2.61)	.3135*	48.72 (20.47)	48.66 (20.40)	48.74 (20.57)	.9831*
Age	≤29	1.34 (2.47)	1.76 (2.81)	1.21 (2.43)	.6790*	47.50 (23.60)	55.73 (16.92)	44.91 (25.16)	.2513*
	30–39	0.51 (0.95)	0.68 (1.14)	0.45 (0.91)	.6593*	52.82 (20.19)	41.89 (7.27)	56.67 (21.99)	.0246*
	40–49	0.63 (1.74)	0.27 (1.01)	0.75 (1.92)	.2345*	44.66 (15.56)	37.73 (10.79)	47.03 (16.33)	.0212*
	50–59	0.50 (1.27)	1.05 (2.21)	0.31 (0.67)	.2726*	56.33 (25.10)	65.32 (23.51)	53.25 (25.21)	.1475*
	60–69	1.05 (3.53)	0.13 (0.63)	1.35 (4.01)	.0186*	54.22 (26.33)	54.89 (26.34)	54.01 (26.52)	.8918*
	≥70	0.89 (2.05)	1.59 (3.19)	0.65 (1.65)	.6057*	50.02 (17.68)	42.26 (12.37)	52.61 (18.85)	.2440*
Hospital type	Tertiary general hospital	0.66 (2.20)	0.49 (1.37)	0.73 (2.48)	.4208*	42.30 (21.56)	43.82 (19.43)	41.63 (22.48)	.5061*
	General hospital	0.85 (2.50)	0.91 (2.30)	0.85 (2.55)	.9311*	38.17 (24.01)	36.39 (26.04)	38.43 (23.83)	.7853*
	Hospital	0.01 (0.11)	0.00 (0.00)	0.01 (0.13)		8.46 (10.63)	7.78 (8.24)	8.67 (11.31)	.5903*
Department	Internal medicine					40.52 (15.72)	41.66 (15.54)	40.14 (15.80)	.5015*
	Other					13.04 (14.52)	11.06 (10.62)	13.68 (15.55)	.1610*

SDM = shared decision-making.

* Paired *t* test.

There was no significant difference in the annual number of outpatient visits between the SDM and non-SDM groups (*P* = .8862). There was no significant difference was observed in sex, age, type of hospital, and department between the 2 groups (*P* = .8610, *P* = .9831, *P* = .2513, *P* = .0246, *P* = .0212, *P* = .1475, *P* = .8918, *P* = .2440, *P* = .5061, *P* = .7853, *P* = .5903, *P* = .5015, *P* = .1610, respectively) (Table 2).

There was no significant difference in the annual number of hospitalization per patient between the SDM and non-SDM groups (*P* = .9543). However, it was significant difference in the hospitalization days per case per patient (*P* = .0044) (Table 3).

**Table 3:** Number of admission per year, hospitalization days per case per patient according to implementation of SDM.

		Admission per year per patient				Hospitalization days per case per patient			
		Total (n = 256)	Patient group (n = 64)	Control group (n = 192)	*P* value	Total (n = 256)	Patient group (n = 64)	Control group (n = 192)	*P* value
Total		1.93 (3.74)	1.91 (4.11)	1.94 (3.62)	.9543*	5.19 (4.66)	3.32 (2.54)	5.62 (4.94)	.0044*
Sex	Female	1.83 (3.91)	2.57 (5.30)	1.59 (3.36)	.4434*	4.86 (3.87)	3.29 (3.40)	5.25 (3.93)	.2140*
	Male	1.98 (3.67)	1.60 (3.47)	2.10 (3.74)	.4215*	5.36 (5.02)	3.34 (2.10)	5.80 (5.37)	.0093*
Age	≤29	2.08 (2.87)	2.59 (3.81)	1.92 (2.61)	.6990*	4.94 (4.75)	2.44 (2.50)	5.62 (5.07)	.1734*
	30–39	1.81 (3.99)	3.89 (7.59)	1.07 (1.29)	.4067*	5.11 (3.94)	4.17 (5.06)	5.47 (3.79)	.7134*
	40–49	1.57 (3.04)	1.08 (2.96)	1.74 (3.09)	.4868*	4.68 (3.15)	2.33 (2.31)	5.07 (3.15)	.1596*
	50–59	2.17 (4.03)	3.61 (5.71)	1.67 (3.23)	.2821*	5.38 (5.37)	3.13 (1.50)	6.08 (5.97)	.0878*
	60–69	1.89 (4.06)	0.52 (1.26)	2.34 (4.54)	.0040*	5.67 (5.64)	3.12 (1.75)	6.02 (5.91)	.0563*
	≥70	2.66 (4.37)	3.29 (5.03)	2.45 (4.36)	.7795*	4.55 (2.77)	5.70 (3.25)	4.09 (2.82)	.6133*
Hospital type	Tertiary general hospital	1.51 (3.23)	1.44 (3.80)	1.53 (2.95)	.8738*	4.68 (4.95)	3.47 (2.83)	4.96 (5.29)	.1640*
	General hospital	1.78 (3.67)	2.82 (4.55)	1.62 (3.52)	.3598*	5.14 (3.90)	3.39 (2.03)	5.46 (4.09)	.0787*
	Hospital	0.18 (1.13)	0.05 (0.29)	0.23 (1.29)	.1420*	2.47 (3.65)	1.00 (NA)	2.62 (3.81)	
Department	Surgery	1.93 (2.71)	2.95 (4.12)	1.72 (2.30)	.2790*	4.67 (5.04)	2.47 (1.53)	5.06 (5.34)	.0103*
	Non surgery (internal medicine)	0.84 (1.95)	0.87 (2.26)	0.83 (1.83)	.8826*	3.39 (3.68)	3.21 (1.83)	3.43 (3.95)	.7572*
	Non surgery (other)	0.52 (1.43)	0.42 (1.00)	0.55 (1.54)	.4943*	1.22 (1.37)	0.82 (0.44)	1.33 (1.53)	.0899*

SDM = shared decision-making.

* Paired *t* test.

There was no significant difference in the direct medical costs, hospitalization costs, outpatient costs, between the SDM and non-SDM groups (*P* = .0995, *P* = .1093, *P* = .3439, respectively) (Table 4).

**Table 4:** Medical cost, phosphorus binding agent, hematopoietic agent prescription rate according to implementation of SDM.

	Total (n = 256)	Patient group (n = 64)	Control group (n = 192)	*P* value
Direct medical cost	31,818 ± 38,790	30,179 ± 19,950	32,363 ± 43,195	.0995*
Admission cost	6945 ± 21,573	4121 ± 12,744	7886 ± 23,753	.1093*
Outpatient cost	24,873 ± 17,217	26,058 ± 7206	24,477 ± 19,442	.3439*
Phosphorous binder	184 (71.88)	41 (64.06)	143 (74.48)	.1486*
Calcium based	110 (42.97)	25 (39.06)	85 (44.27)	.5598*
Non-calcium based	124 (48.44)	28 (43.75)	96 (50.00)	.4703*
Sevelamer	114 (44.53)	27 (42.19)	87 (45.31)	.7715*
Lanthanium	9 (3.52)	1 (1.56)	8 (4.17)	.5567*
Sucroferric oxyhydroxide	21 (8.20)	1 (1.56)	20 (10.42)	.0486*
Hematopoietic agent	231 (90.23)	57 (89.06)	174 (90.62)	.9032*
Erythropoietin	83 (32.42)	23 (35.94)	60 (31.25)	.5895*
Darbepoetin	43 (16.80)	6 (9.38)	37 (19.27)	.1008*
CERA	105 (41.01)	28 (43.75)	77 (40.10)	>.99*

SDM = shared decision-making.

* Paired *t* test.

No significant differences were observed in the prescription rates of phosphate binders and hematopoietic agents between the SDM and non-SDM groups (*P* = .1486, *P* = .5598, *P* = .4703, *P* = .7715, *P* = .5567, respectively). However, the prescription rate of the phosphate binder sucroferric oxyhydroxide was higher in the non-SDM group (*P* = .0486) (Table 4).

There was no significant difference in the hematopoietic agent type (erythropoietin, dabepoetin, continuous erythropoietin receptor activator) between 2 groups (*P* = .9032, *P* = .5895, *P* = .1008, *P* > .99, respectively) (Table 4).

Based on the above results, *P* values < .05, including hospitalization days and sucroferric oxyhydroxide prescriptions. Among the results, the *P* value of hospitalization days was .004 and was considered significant.

## 4. Discussion

In this study, it was confirmed that there is an effect of reducing hospitalization days when patients who participated in the pilot project for home management of PD decided on PD through the SDM process. These results can serve as a basis for applying the SDM process when patients decide on PD in the future. Although this study was applied only to PD patients, future research can be expanded to investigate whether the SDM process is also effective in the decision-making process for HD.

Since PD allows patients to perform dialysis at home, it can be an alternative to HD for patients who find it difficult to visit the hospital frequently (e.g., those living in geographically restricted areas such as islands or mountainous regions or those with occupational characteristics that make hospital visits challenging). Typically, patients need to visit the hospital once a month to ensure that PD is being performed effectively. Since this study confirmed the effect of reducing hospitalization days for PD patients who underwent the SDM process, it can be beneficial for patients who find long-term hospitalization difficult due to medical expenses or livelihood concerns.

Recently, by using automated PD that automatically exchanges dialysis fluid during sleep, patients can use their daytime hours freely. Additionally, with the installation of a digital patient management platform, patients’ dialysis treatment data is automatically stored and transmitted to healthcare providers, allowing the patient’s condition and treatment outcomes to be monitored through the digital platform without the need for frequent hospital visits.

There are other studies on the SDM process where doctors and patients make decisions together. In a randomized study of 363 patients, the SDM process helped improve the quality of care and clarify treatment preferences for older adults with CKD over 6 months.^[10]^ Additionally, a meta-analysis study of 15 patients who received PD for <12 months highlighted the importance of SDM with medical staff.^[13]^ Furthermore, a prospective observational study conducted at 31 medical institutions in Korea until July 2012 on 1200 patients who started dialysis CRC showed that the mortality rate more than doubled during the follow-up period of more than 2 years.^[2]^ These results suggest that the SDM process not only impacts the method of dialysis but also the lifelong dialysis management through ongoing communication between the patient and healthcare provider after the start of dialysis.

Although there are currently no home management services being implemented overseas, various countries are making efforts to increase the proportion of home dialysis (mainly PD) due to the rising medical expenses for dialysis patients.^[25]^ Thailand has adopted a PD-first policy, providing insurance coverage for those choosing PD, and only supporting HD costs for patients unable to undergo PD.^[26]^ Taiwan has implemented multidisciplinary pre-dialysis education to activate PD, which has also contributed to improving patient survival rates.^[27–29]^ In Australia, incentives are provided for home dialysis, covering the costs of water, sewage, and electricity needed for home dialysis. New Zealand prefers home dialysis without financial incentives or policies, based on the clinical superiority of home dialysis. They are making efforts to improve clinical outcomes by implementing patient education, selection, and guideline-based treatment to reduce PD failures.^[30,31]^ In Ontario, Canada, more generous funding for home dialysis than hospital HD has increased the proportion of home dialysis, particularly PD.^[32]^ The United States has attempted financial incentives for home HD and PD by shifting from a fee-for-service system to a prospective payment system to reduce medical expenses. Additionally, policies like the AAKHI aim to have 80% of new ESRD patients undergo home dialysis or kidney transplants by 2025 to further reduce healthcare costs.^[33]^ Spain has been implementing SDM for pre-dialysis patients to increase PD use and providing continuous education on PD to nephrology fellows and healthcare professionals.^[34]^ Japan is implementing SDM to increase the use of PD and provides higher reimbursement to institutions with PD experience.^[35]^ The results of PD policies and education overseas show that the SDM process for pre-dialysis patients is not only directly related to improving patient education satisfaction but also survival rates. To encourage patient participation in the decision-making process for choosing a dialysis method, the active use of patient education programs and SDM is essential. To incentivize the participation of healthcare professionals, active measures such as rewards or incentives for the education process, as seen in overseas cases, are necessary. Although it is unclear whether the higher conversion rate in the non-SDM group was due to disease progression, PD complications, or other factors due to limitations of this study, further investigation into this cause in the future would provide further insight into the effect of SDM on patient selection and outcomes.

This study’s strength lies in its focus on SDM for PD, revealing that SDM can reduce hospitalization days, which may lower costs and improve patient satisfaction. However, the study showed no significant effects of SDM on mortality, emergency room visits, or HD conversion. With a limited sample size and single-country scope, generalizability is restricted. While findings are applicable in similar healthcare systems, they may not extend broadly due to regional differences in health policies, PD support, and patient demographics. Limitations of this study include the lack of adjustment for key variables that can significantly influence outcomes in PD patients. While mortality and HD transfer were investigated, other critical factors, such as serum albumin levels, various biomarkers, cause of end-stage kidney disease, residual kidney function, and social status and support, were not accounted for. These unadjusted variables may have impacted the study’s results, potentially confounding the relationship between SDM and the outcomes examined. Future studies should consider these factors to more accurately assess the effects of SDM on PD patient prognosis.

The Korean Society of Nephrology is currently conducting an “SDM campaign” where patients choose a dialysis method, a shared doctor program is developed, and research results and proposals for a new Korean-style treatment decision-making process are presented.^[36]^ Since December 2019, the Korean Society of Nephrology has been promoting a pilot project for the home management of PD patients to reduce unnecessary medical expenses and improve patient quality of life. If the results of this pilot project, such as the expansion of application to inpatient care, the confirmation of insurance coverage for home management services, the adjustment of standards, the expansion of the number of educational consultation fees, and the establishment of a separate insurance fee for SDM, are reflected, more efficient services can be provided. It is hoped that the results of this study will serve as a basis for the actual implementation of home management services for PD patients in the future.

## Author contributions

**Conceptualization:** Ho Sik Shin, Jinseog Kim, Jeonghwan Lee, Ji Hyeon Park, Gang Jee Ko, Won Min Hwang, Do Hyoung Kim, Young Ki Lee.

**Data curation:** Ho Sik Shin, Jinseog Kim, Jeonghwan Lee, Ji Hyeon Park, Gang Jee Ko, Won Min Hwang, Do Hyoung Kim, Young Ki Lee.

**Formal analysis:** Ho Sik Shin, Jinseog Kim, Jeonghwan Lee, Ji Hyeon Park, Gang Jee Ko, Won Min Hwang, Do Hyoung Kim, Young Ki Lee.

**Funding acquisition:** Ho Sik Shin, Jinseog Kim, Jeonghwan Lee, Ji Hyeon Park, Gang Jee Ko, Won Min Hwang, Do Hyoung Kim, Young Ki Lee.

**Investigation:** Ho Sik Shin, Jinseog Kim, Jeonghwan Lee, Ji Hyeon Park, Gang Jee Ko, Won Min Hwang, Do Hyoung Kim, Young Ki Lee.

**Methodology:** Ho Sik Shin, Jinseog Kim, Jeonghwan Lee, Ji Hyeon Park, Gang Jee Ko, Won Min Hwang, Do Hyoung Kim, Young Ki Lee.

**Project administration:** Ho Sik Shin, Jinseog Kim, Jeonghwan Lee, Ji Hyeon Park, Gang Jee Ko, Won Min Hwang, Do Hyoung Kim, Young Ki Lee.

**Resources:** Ho Sik Shin, Jinseog Kim, Jeonghwan Lee, Ji Hyeon Park, Gang Jee Ko, Won Min Hwang, Do Hyoung Kim, Young Ki Lee.

**Software:** Ho Sik Shin, Jinseog Kim, Jeonghwan Lee, Ji Hyeon Park, Gang Jee Ko, Won Min Hwang, Do Hyoung Kim, Young Ki Lee.

**Supervision:** Ho Sik Shin, Jinseog Kim, Jeonghwan Lee, Ji Hyeon Park, Gang Jee Ko, Won Min Hwang, Do Hyoung Kim, Young Ki Lee.

**Validation:** Byung Hwa Park, Ho Sik Shin, Jinseog Kim, Jeonghwan Lee, Ji Hyeon Park, Gang Jee Ko, Won Min Hwang, Do Hyoung Kim, Young Ki Lee.

**Visualization:** Ho Sik Shin, Jinseog Kim, Ji Hyeon Park, Gang Jee Ko, Won Min Hwang, Do Hyoung Kim, Young Ki Lee.

**Writing – original draft:** Byung Hwa Park, Ho Sik Shin.

**Writing – review & editing:** Byung Hwa Park, Ho Sik Shin.

## Supplementary Material


