# Supplementary material for: Effects of shared decision-making on the prognosis of peritoneal dialysis patients
Source: Medicine (Baltimore). 2024 Nov 22;103(47):e40659. doi: 10.1097/MD.0000000000040659 (PMC11596416; doi:10.1097/MD.0000000000040659)
Supplement: Supplementary file 1 [file medi-103-e40659-s001.docx]

1. Catholic Kwandong University Eunpyeong St. Mary's Hospital

2. Kyung Hee University Medical Center, Gangdong

3. Konkuk University Medical Center

4. Korea University Guro Hospital

5. Seoul National University Hospital

6. Asan Medical Center

7. Seoul Metropolitan Boramae Hospital

8. Soonchunhyang University Hospital Seoul

9. Korea University Anam Hospital

10. Seoul St. Mary's Hospital, The Catholic University of Korea

11. Severance Hospital, Yonsei University College of Medicine

12. Hallym University Kangnam Sacred Heart Hospital

13. Kosin University Gospel Hospital

14. Daedong Hospital

15. Dongrae Bong Seng Hospital

16. Pusan National University Hospital

17. Kim Woon Mook Memorial Bong Seng Hospital, Jeonghwa Medical Foundation

18. Haeundae Paik Hospital, Inje University

19. Busan Paik Hospital, Inje University

20. Maryknoll Hospital, Catholic Diocese of Busan Foundation

21. Kyungpook National University Hospital

22. Dongsan Hospital, Keimyung University

23. Daegu Catholic University Medical Center

24. Daegu Fatima Hospital

25. Yeungnam University Medical Center

26. Chilgok Kyungpook National University Hospital

27. Incheon St. Mary's Hospital, The Catholic University of Korea

28. Inha University Hospital

29. Konyang University Hospital

30. Daejeon Eulji Medical Center, Eulji University

31. Daejeon Sun Hospital, Yeonghoon Medical Foundation

32. Ulsan University Hospital

33. Uijeongbu St. Mary's Hospital, The Catholic University of Korea

34. Korea University Ansan Hospital

35. National Health Insurance Service Ilsan Hospital

36. Myongji Hospital

37. Seoul National University Bundang Hospital

38. Soonchunhyang University Bucheon Hospital

39. Ilsan Paik Hospital

40. CHA Bundang Medical Center, CHA University

41. Hallym University Dongtan Sacred Heart Hospital

42. Hallym University Sacred Heart Hospital

43. Chuncheon Sacred Heart Hospital, Hallym University

44. Chungbuk National University Hospital

45. Dankook University Hospital

46. Soonchunhyang University Cheonan Hospital

47. Mokpo Jung-ang Hospital, Mokpo Gu-am Medical Foundation

48. Soonchunhyang University Gumi Hospital

49. Pohang Semyung Christian Hospital, Hansung Foundation

50. Gyeongsang National University Hospital

51. Yangsan Pusan National University Hospital

52. Changwon Fatima Hospital, Benedictine Sisters of Daegu Foundation

53. Changwon Gyeongsang National University Hospital

54. Samsung Changwon Hospital, Sungkyunkwan University Foundation

55. Kangbuk Samsung Hospital

56. Kyung Hee University Hospital

57. Samsung Medical Center (Seoul)

58. Yonsei University College of Medicine Gangnam Severance Hospital

59. Chung-Ang University Hospital

60. Korea Veterans Hospital, Central Veterans Hospital of Korea

61. Nowon Eulji Medical Center, Eulji University Hospital

62. Seongnam Medical Foundation Kangdong Sacred Heart Hospital

63. Ajou University Hospital

64. Catholic University of Korea Bucheon St. Mary's Hospital

65. Dongguk University Ilsan Buddhist Hospital

66. Seongnam City Medical Center

67. Yonsei University College of Medicine Yongin Severance Hospital

68. Namchon Medical Foundation Siheung Hospital

69. Eulji University Hospital, Eulji Foundation, Uijeongbu Eulji University Hospital

70. Hanyang University Guri Hospital

71. Dong-A University Hospital

72. Yeongjae Medical Foundation Keunsol Hospital

73. Gil Medical Foundation Gil Hospital

74. Incheon Sejong Hospital

75. Nazareth Medical Foundation Nazareth International Hospital

76. Catholic University of Korea Daecheon St. Mary's Hospital

77. Chungnam National University Hospital

78. Yonsei University Wonju Severance Christian Hospital

79. Mokpo City Medical Center

80. Chonbuk National University Hospital

81. Medical Foundation Andong Hospital

82. Chosun University Hospital

83. Jeju Hanla Hospital
