# Supplementary material for: Effects of shared decision-making on the prognosis of peritoneal dialysis patients
Source: Medicine (Baltimore). 2024 Nov 22;103(47):e40659. doi: 10.1097/MD.0000000000040659 (PMC11596416; doi:10.1097/MD.0000000000040659)
Supplement: Supplementary file 2 [file medi-103-e40659-s002.docx]

In SDM measurement scale, on a 5-point scale, the proportion of 4 or more (yes, very much yes) was identified. The response rate of 4 points or more (positive answers) in 5 out of 9 items was more than 90%, and SDM was effective in deciding on dialysis treatment. The contents of the questionnaire are as follows.

1) My doctor made it clear that a decision needed to be made

2) My doctor wanted to know exactly how I was involved in making decisions

3) My doctor told me that there are several options depending on my health condition

4) My doctor explained the pros and cons of each treatment in detail

5) My doctor helped me understand the information

6) My doctor asked me what my preferred treatment was

7) My doctor and I thoroughly evaluated the different treatments

8) My doctor and I chose a treatment together

9) My doctor and I agreed on a course of treatment

In patient satisfaction scale, on a 5-point scale, the proportion of 4 or more (yes, very much yes) was identified. The response rate of 4 points or more (positive answers) in 4 out of 6 items was more than 90%, and the patient showed satisfactory results in the process of determining the dialysis method through the implementation of SDM. The contents of the questionnaire are as follows.

1) My doctor helped me understand all the information

2) My doctor knew what was important to me

3) My doctor answered all my questions

4) I was fully involved in the process of deciding on my treatment

5) I decided about further treatment with my primary care physician

6) I am satisfied with the way my treatment was discussed and decided

In disease perception scale, on a 5-point scale, the proportion of 4 or more (yes, very much yes) was identified. Among the 10 items, the proportion of responses with a score of 4 or more in items 7, 8, and 10, which are positive indicators, was high at 51.3%, 46.8%, and 57.6%, respectively, while the rest of the negative indicators were relatively low. Therefore, the patients who participated in the educational counseling to determine the type of dialysis had high positive perceptions and low negative perceptions, so it can be evaluated that joint decision-making was relatively effective and well-executed. The contents of the questionnaire are as follows.

1) There's little I can do to improve my illness

2) There is nothing that can help my condition

3) My actions will have no effect on the outcome of my illness

4) No matter what I do, my illness won't change

5) My illness will last for a short time

6) My illness makes it hard for those around me

7) My treatment can control my illness

8) The negative effects of my illness can be prevented or avoided with my own treatment

9) I don't understand my illness

10) There are many things I can do to control the symptoms of my illness

The effect of SDM on dialysis method selection, on a 5-point scale, the proportion of 4 or more (yes, very much yes) was identified. 93 out of 101 patients (92.1%) who received educational counseling to confirm the type of dialysis said that the implementation of SDM helped them choose a dialysis method.

The contents of list are belows.

1. **Health-Related**

- I want to protect my heart.

- I want to protect my bones, joints, and nerves.

- I want to live according to my will.

- I don't want to be a burden to my family.

- I want to maintain a high quality of life as much as possible.

- I want to spend a day without the burden of dialysis.

- I don't want to undergo dialysis every day.

- I want to receive regular medical check-ups.

- I want to be able to control my daily life myself.

- I live a regular life.

- I plan and act on a daily basis.

- I enjoy exercising.

2. **Dialysis Environment**

- I want to go to the hospital as little as possible.

- I prefer professional dialysis.

- I like things that are familiar.

- I dislike changes in my environment.

- I like experiencing new things.

- I enjoy watching TV.

- It is difficult for me to lie down for a long time.

- I am afraid of needles.

- I am very conscious of others' opinions.

- I rely heavily on my spouse in my daily life.

- I need someone to take care of me.

- I don't want a dialysis machine in my house.

3. **Daily Life**

- I want to spend as much time with my family as possible.

- I live with elderly parents who need care.

- I am raising children.

- I need to go to school or work.

- I want to eat and drink what I like.

- I sweat a lot, so I take frequent baths.

- I care about my appearance.

- It takes me a long time to get used to new things.

- I don't have anyone to take care of me.

- I love traveling and cannot give it up.

- I travel abroad frequently.
